# Effect of modern high-dose versus standard-dose radiation in definitive concurrent chemo-radiotherapy on outcome of esophageal squamous cell cancer: a meta-analysis

**DOI:** 10.1186/s13014-019-1386-x

**Published:** 2019-10-17

**Authors:** He-San Luo, He-Cheng Huang, Lian-Xing Lin

**Affiliations:** Department of Radiation Oncology, Shantou Central Hospital, Affiliated Shantou Hospital of Sun Yat-sen University, NO. 114 Waima Road, Shantou, Guangdong China

**Keywords:** High-dose, Standard-dose, Chemo-radiotherapy, Esophageal squamous cell cancer, Meta-analysis

## Abstract

**Background and objectives:**

Radiation Therapy Oncology Group (RTOG) 94–05 has demonstrated that higher dose radiation didn’t improve outcome of patients with esophageal cancer (EC). However, several retrospective studies showed that a higher dose radiation based on modern radiotherapy techniques could improve overall survival (OS) and local control rate (LCR) of patients with EC, especially esophageal squamous cell cancer (ESCC). As trials have provided updated and controversial data, we performed this updated meta-analysis to investigate whether high-dose (> = 60 Gy) radiotherapy in definitive concurrent chemo-radiotherapy (CCRT) could yield benefit compared to standard dose radiotherapy.

**Methods:**

A systematic literature search was carried out in the database of MEDLINE, PubMed and Embase. All studies published between 1 January 1990 and 31 December 2018 on the association between radiation dose and curative efficiency in EC were included in this meta-analysis. The hazard ratio (HR) was used to evaluate the time-to-event data employing RevMan version 5.3.

**Results:**

Eight articles with a total of 3736 patients were finally included. Results indicated that there was a significant benefit in favor of high dose radiotherapy (HD-RT) regarding OS (HR = 0.78, 95%CI: 0.72–0.84, *p* < 0.001; 2-year OS risk ratio (RR) = 1.25, 95%CI: 1.14–1.37, *p* < 0.001), progression-free survival (PFS) (*P* = 0.001, HR = 0.7, 95%CI: 0.57–0.87) and LRFS (*P* < 0.001, HR = 0.52, 95%CI: 0.36–0.74) .

**Conclusions:**

HD-RT (> = 60 Gy) based on modern radiotherapy techniques in definitive CCRT appears to improve OS, PFS amd LRFS compared to the SD-RT in patients with ESCC.

## Introduction

Esophageal cancer (EC) is one of the most common malignant tumors and the fourth most common cause of cancer-related deaths worldwide [1, 2]. Approximately more than a half of the total cases occur in China [1]. In China, squamous cell cancer (SCC) is the most common type of EC, accounting for about 90% of all the patients with EC [3]. For early EC, surgery is the main curative treatment modality. However, most patients are not diagnosed until the disease is at an advanced stage [4].

For patients with locally advanced inoperable disease or patients refused surgery, definitive concurrent chemoradiotherapy (CCRT) is recommended as a standard treatment modality based on the results of the Intergroup Radiation Therapy Oncology Group (RTOG)-8501 which improved the local control (LC) and overall survival (OS) with CCRT compared with radiotherapy (RT) alone [5]. The optimal radiation dose of CCRT was subsequently explored by the landmark RTOG 94–05 randomized controlled trial (RCT) and the interim analysis showed dose escalation from 50.4 to 64.8 Gy did not increase OS or local regional control [6]. Since then, 50.4 Gy has become the accepted standard dose for EC patients undergoing CCRT in Europe and American guidelines. Nevertheless, no consensus has been reached globally on the appropriate radiation dose of definitive CCRT for EC. More than a half of patients treated with standard-dose CRT were eventually developed recurrence or distant metastases and succumbed to this disease [7]. A recent systematic review has performed a pooled analysis to investigate whether high-dose radiotherapy (HD-RT) could improve LC or OS and found that HD-RT improved clinical outcomes as compared to standard-dose radiotherapy (SD-RT) [8]. As several studies have indicated that a higher dose above 50.4Gy of CCRT could be safely administered without significant untoward effects and yield high probability of LC [9–11], a dose of 60.0 Gy or more has become a more popular dose of CCRT in Asian countries, where ESCC is the predominant histological type. However, There is no prospective clinical trial to investigate the role of HD-RT based on modern radiation techniques on the prognosis of ESCC patients.

In this study, we performed an updated meta-analysis to evaluate whether a higher dose above 60Gy of CCRT could improve the prognosis of ESCC patients as compared to a standard dose.

## Patients and methods

### Search strategy, studies identification and selection

A systematic literature search was carried out in the database of MEDLINE, PubMed and Embase. All studies published between 1 January 1990 and 31 December 2018 on the association between radiation dose and curative efficiency in EC were considered in this meta-analysis. The following terms were used for search: (“esophageal”[Title]) or (“oesophageal”[Title]) or (“esophagus”[Title])) and (“tumor”[Title]) or (“cancer”[Title]) or (“carcinoma”[Title]) or (“neoplasm”[Title]) or (“neoplasms”[Title])) and (“chemoradiation”[Title]) or (“chemoradiotherapy”[Title]) or (“radiochemotherapy”[Title]) or (“chemo-irradiation”[Title]) or (“chemo-radiotherapy”[Title])) and (“dose” [Abstract]). Inclusion criteria were defined as follows: 1) Studies on patients with esophageal cancer treated with concurrent chemoradiotherapy. 2) Studies comparing the curative efficiency in EC patients who were treated with high dose radiotherapy (HD-RT) or standard dose radiotherapy (SD-RT) (HD-RT was considered > = 60 Gy of radiation dose and SD-RT was considered 45–59.4 Gy of radiation dose). 3) Studies included most patients with esophageal squamous cell carcinoma. Studies were excluded as following: 1) Neoadjuvant or adjuvant chemoradiotherapy combined with surgery. 2) Radiotherapy was delivered by Co-60 or by unconventional fractions. 3) Review or case report, with other sites of cancers, and meta-analysis. 4) Results mixed with HD-RT and SD-RT, results not reported exactly. The search did not restrict the type of publication or periodical, but limit to English language.

### Quality assessment

The quality of included studies was independently assessed by two assessors. The 9-star Newcastle-Ottawa Scale (Available from: http://www.ohri.ca/programs/clinical_epidemiology/oxford.htm) was adopted for assessment of the non-randomized studies. The quality categories were defined as follows: high quality (score 7–9), medium quality (score 4–6) and low quality (score less than 4). Quality of RCT was assessedusing the 7-point JADAD scale.

### Endpoints of interest

We collected the information about OS, 2-year survival rate, progression-free survival (PFS) and local recurrence-free survival (LRFS) from the included studies. Patients were divided into two groups according radiation dose threshold as defined by the individual studies.

### Data extraction and synthesis

**Fig. 1:**
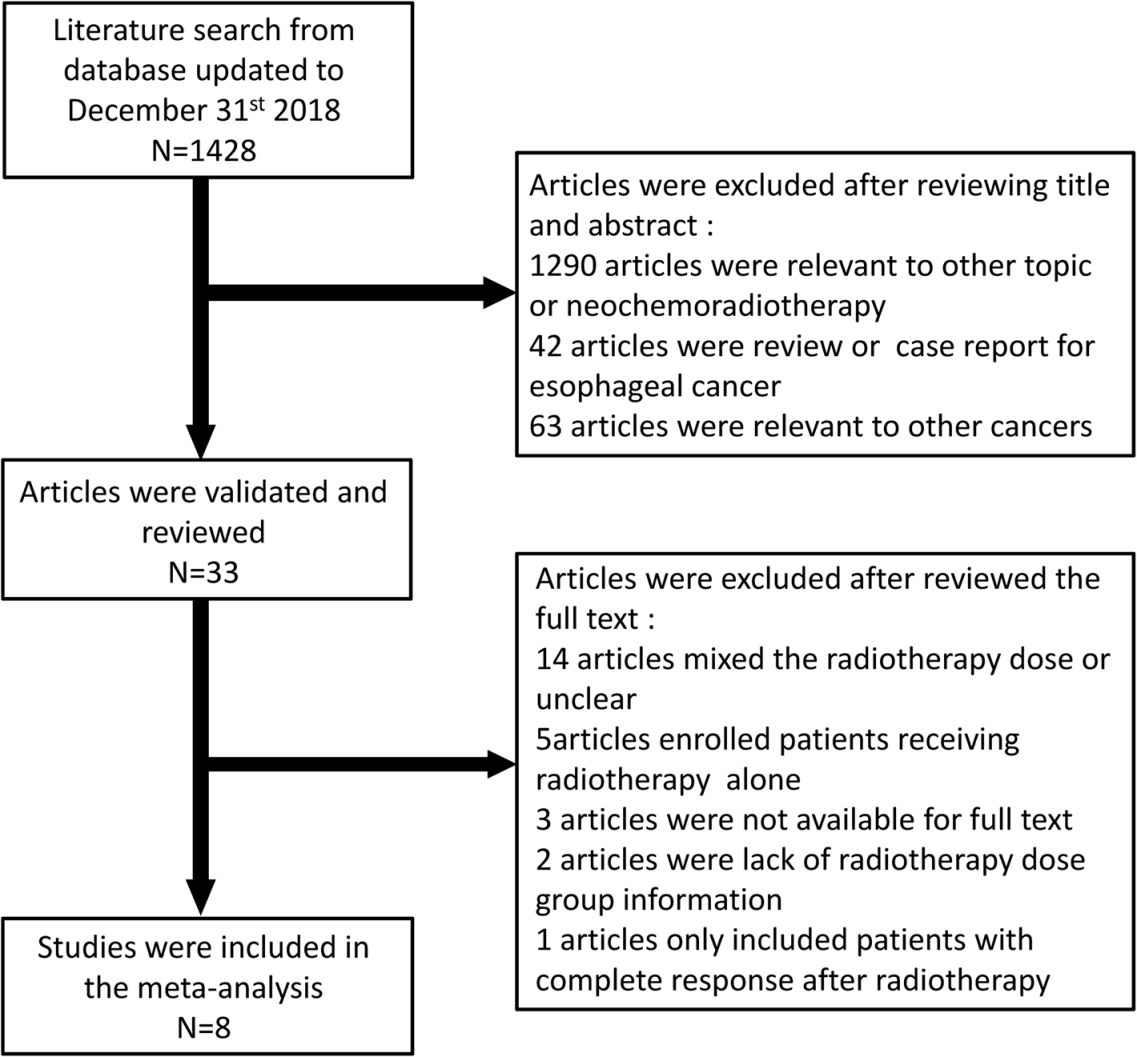
Literature search strategy and study selection for the meta-analysis

The abstract of each study was reviewed by HS Luo and irrelevant or overlapping studies were removed according to the criteria mentioned above, thus creating a preliminary set of potentially relevant publications (Fig. 1). Then, two authors (HC Huang and LX Lin) independently reviewed the full articles to exclude studies unqualified and extracted data from all included studies regarding the first author, country, year of publication, study period, type of study, sample size, age, clinical stage, pathological types, chemotherapy regimens, radiation technology, and radiation dose in HD-RT and SD-RT groups. The evaluation results were compared and re-evaluated until consensuses were reached between two authors. The frequencies of LRFS and PFS from the different groups were expressed as an OR with its 95% CI. If a figure for HR and 95% CI was not available, an estimate value was calculated indirectly by using the methods described by Tierney et al. Survival rates from Kaplan-Meier curves were read using Engauge Digitizer version 4.1 (available from: http://digitizer.sourceforge.net/) and the resulting data were then entered in the calculation spreadsheet appended to Tierney’s paper.

### Statistical analysis

All analyses were performed using Review Manager Version 5.3. Statistical heterogeneity among various studies was tested using I2-statistic. Fixed-effects model was used for risk ratio (RR) and hazard ratio (HR) analysis if there was no statistical heterogeneity (I2 < 50%, *P* ≥ 0.1) among studies, otherwise random-effects model would be used. Forest plots were generated to show the estimated RRs and HRs, representing the theoretical gain in absolute percentage on the basis of OS, 2-year survival rate, LRFS, and PFS in the included trials. Upper limit and lower limit of 95% confidence intervals (CIs) were calculated. The tests were considered statistically significant if *P* values were less than 0.05. All the P values were two sided. Funnel plots were used to assess the potential of publication bias.

### Grading of Recommendations Assessment, Development and Evaluation (GRADE) assessment

We followed the GRADE approach to assess the quality of the evidence from this meta-analysis. In this approach, guidelines apply a set of predetermined domains that either increase or decrease the level of confidence in the evidence. Domains that reduce confidence in the evidence are: risk of bias, inconsistency of results, indirectness of evidence, imprecision, and publication bias. On the other hand, a large magnitude of effect, confounding that increases effect magnitude and a dose-response gradient can increase confidence. Two researchers discussed the domains for each outcome until consensus was reached.

## Results

### Eligible studies

We identified 1428 potential relevant articles from electronic databases according the defined search strategy. After layer of screening by examination of the titles, abstracts, and the full texts, 1420 articles were excluded according the inclusion criteria and exclusion criteria. As a result, eight articles, with a total of 3736 patients, were included in the final meta-analysis (Fig. 1).

### Characteristics of included studies

The detailed characteristics of these included studies were summarized in Table 1 [6, 12–18]. One RCT (RTOG 94–05) and seven retrospective studies comprised the population of the meta-analysis. There were six studies from Asian country (including two from Korea, one from Taiwan area and three from China) and two studies from western country (including one from USA and one from France). All the patients were treated with concurrent chemo-radiotherapy. The majority of the enrolled patients were pathologically diagnosed as esophageal squamous cell carcinoma (3660/3736, 98.0%). Most of the patients received modern radiotherapy (3D-conformal RT or IMRT). The total delivered radiation dose ranged from 45 to 75.6 Gy. All eight studies provided information on overall survival. Four studies reported the PFS and two studies reported LRFS. HR and its 95% CI were estimated based on the Kaplan-Meier curves of patients receiving the assigned different dose of radiation. The thresholds between HD-RT and SD-RT from each study were mainly around 60 Gy.

**Table 1:** Characteristics of studies included in meta-analysis

Author	Geographic area	Year	Study period	Study design	No.of patients	Gender(M/F)	Median age(y)	Clinical Stage	Histology types (SCC/Other)	Chemotherapy regimens	Radiation technology	Radiation Dose groups	Quality
Minsky [6]	USA	2002	1995–1999	Prospective (Phase III)	218	154/64	64	II-III	187/31	PF	3DRT	64.8Gy 50.4Gy	5
Clavier [12]	France	2011	2003–2006	Retrospective	143	118/25	–	I-IVa	113/30	PF	3DRT	66 Gy 50Gy	7
Suh [13]	Korea	2014	1998–2008	Retrospective	126	117/9	66	II-III	117/9	PF	3DRT	>60Gy(60–75.6Gy) <=60Gy(45–59.4Gy)	7
Chen [14]	China	2016	2008–2013	Retrospective	648	619/29	–	I-IV	648/0	CCRT	3D-IMRT	>60Gy 50.4Gy	8
Kim [15]	Korea	2017	1994–2013	Retrospective	236	226/10	66	II-III	230/6	PF	3D-IMRT	> = 60Gy <60Gy	8
Deng [16]	China	2017	2010–2014	Retrospective	139	95/44	67	I-III	139/0	TPF	3D-IMRT	> 59.4Gy 50.4Gy	8
Chang [17]	Taiwan area	2017	2006–2014	Retrospective	2061	1958/103	57	I-III	2061/0	CCRT	IMRT	> = 60Gy(60-72Gy) <60Gy(45–59.4Gy)	7
Chen [18]	China	2018	2004–2013	Retrospective	165	139/26	55	I-IV	165/0	CCRT	2/3D-IMRT	<60Gy(56–60) 60-65Gy >65Gy	5

*M* Male, *F* Female, *SCC* Squamous cell cancer, *3DRT* Three dimensional conformal radiotherapy, *IMRT* Intensity-modulated radiotherapy, *PF* Cisplatin + 5-fluorouracil, *CCRT* Concurrent chemo-radiotherapy, *TPF* Taxane+cisplatin+ 5-fluorouracil

### Effect of radiation dose on overall survival

**Fig. 2:**
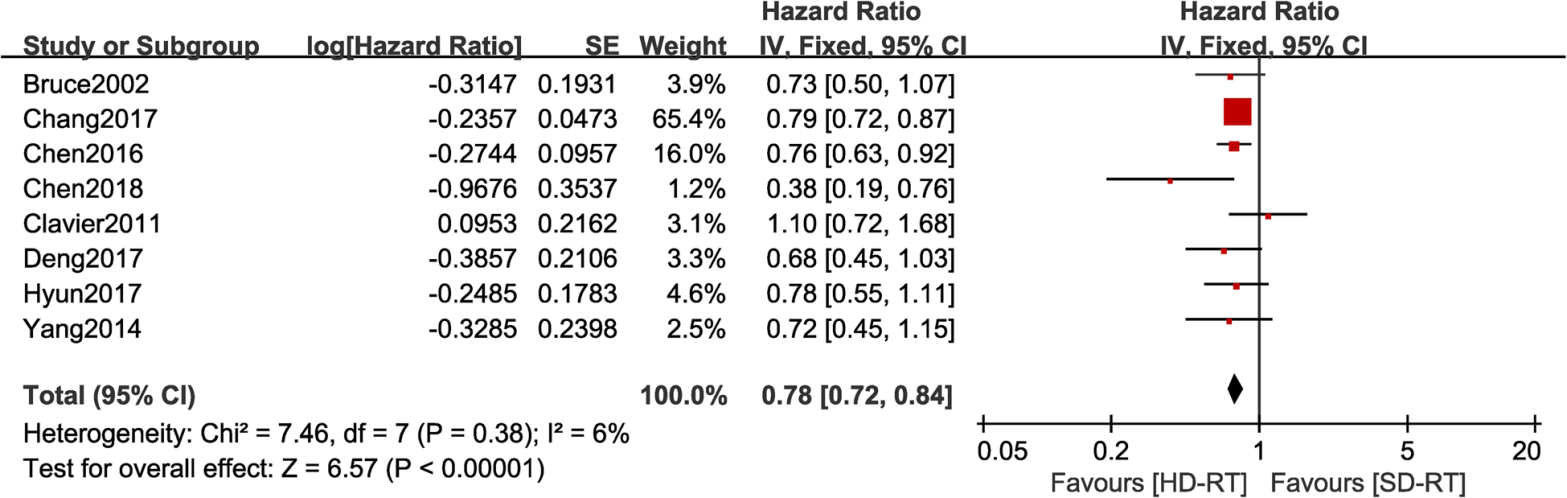
Forest plot of hazard ratio of overall survival (OS) between high dose radiotherapy (HD-RT) and standard dose radiotherapy (SD-RT) in EC patients treated with chemo-radiotherapy

**Fig. 3:**
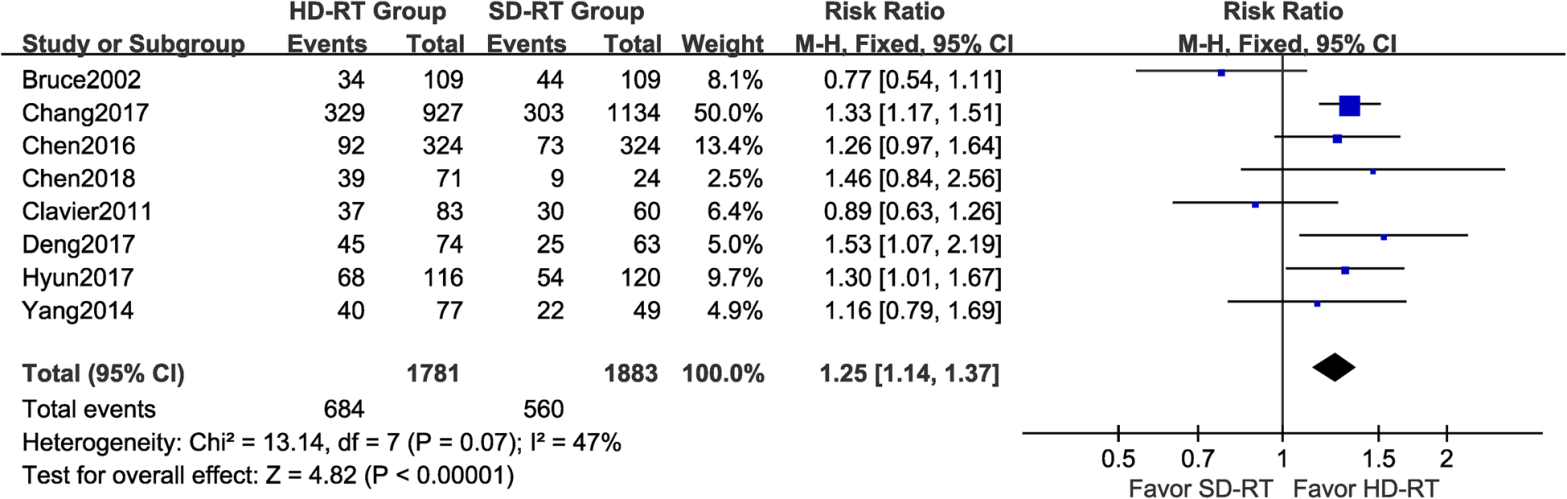
Forest plot of risk ratio (RR) of 2-year overall survival (OS) rate between high dose radiotherapy (HD-RT) and standard dose radiotherapy (SD-RT) in EC patients treated with chemo-radiotherapy

Eight articles reported overall survival curve of patients in HD-RT and SD-RT groups. There was no significant heterogeneity for the results among these studies (*P* = 0.38, I2 = 6%) that a fixed-effects model was used for further analysis. As shown in Fig. 2, patients in HD-RT group had overall survival benefit when compared with patients in SD-RT group (pooled HR = 0.78, 95%CI: 0.72–0.84, *p* < 0.001). In addition, as shown in Fig. 3, benefit in 2-year overall survival rate was gained by patients in HD-RT group (pooled RR = 1.25, 95%CI: 1.14–1.37, *p* < 0.001). A fix-effects model was applied in this analysis because of no significant heterogeneity among these studies (*P* = 0.07, I2 = 47%).

### Effect of radiation dose on PFS

**Fig. 4:**
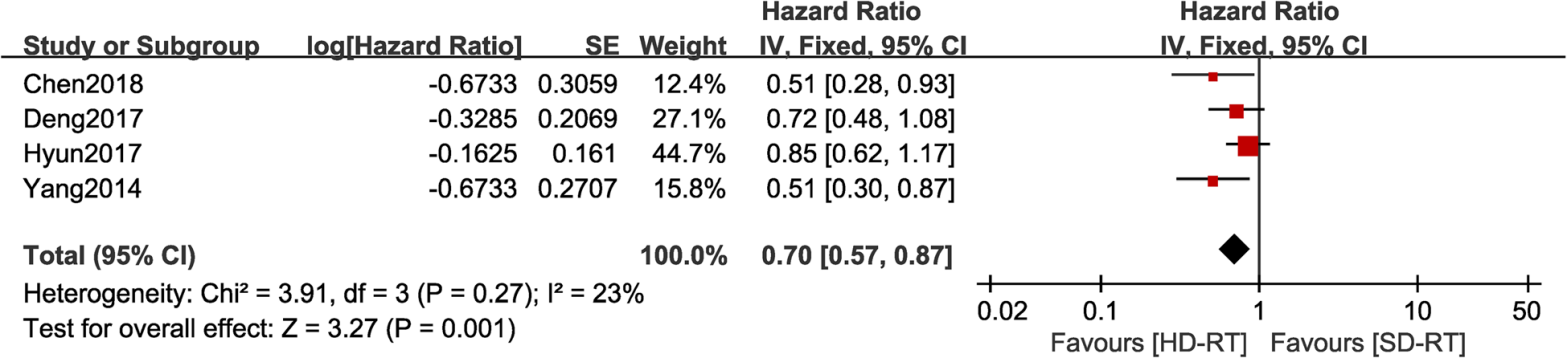
Forest plot of hazard ratio of progression-free survival (PFS) between high dose radiotherapy (HD-RT) and standard dose radiotherapy (SD-RT) in EC patients treated with chemo-radiotherapy

PFS data was extracted from four studies including 594 patients. There was no significant heterogeneity among these studies (*P* = 0.27, I2 = 23%), hence a fixed-effects model was used for pooled analysis. As shown in Fig. 4, PFS of patients in HD-RT group was significantly better than that in SD-RT (*P* = 0.001, HR = 0.7, 95%CI: 0.57–0.87).

### Effect of radiation dose on LRFS

**Fig. 5:**
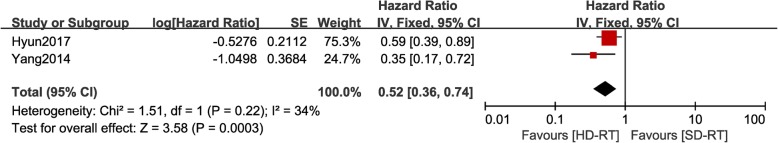
Forest plot of hazard ratio of local recurrence-free survival (LRFS) between high dose radiotherapy (HD-RT) and standard dose radiotherapy (SD-RT) in EC patients treated with chemo-radiotherapy

LRFS data was reported in two studies including 362 patients. There was no significant heterogeneity between the two studies (*P* = 0.22, I2 = 34%), hence a fixed-effects model was used for pooled analysis. As shown in Fig. 5, LRFS of patients in HD-RT group was significantly better than that in SD-RT (*P* < 0.001, HR = 0.52, 95%CI: 0.36–0.74).

### Publication bias and GRADE assessment

**Fig. 6:**
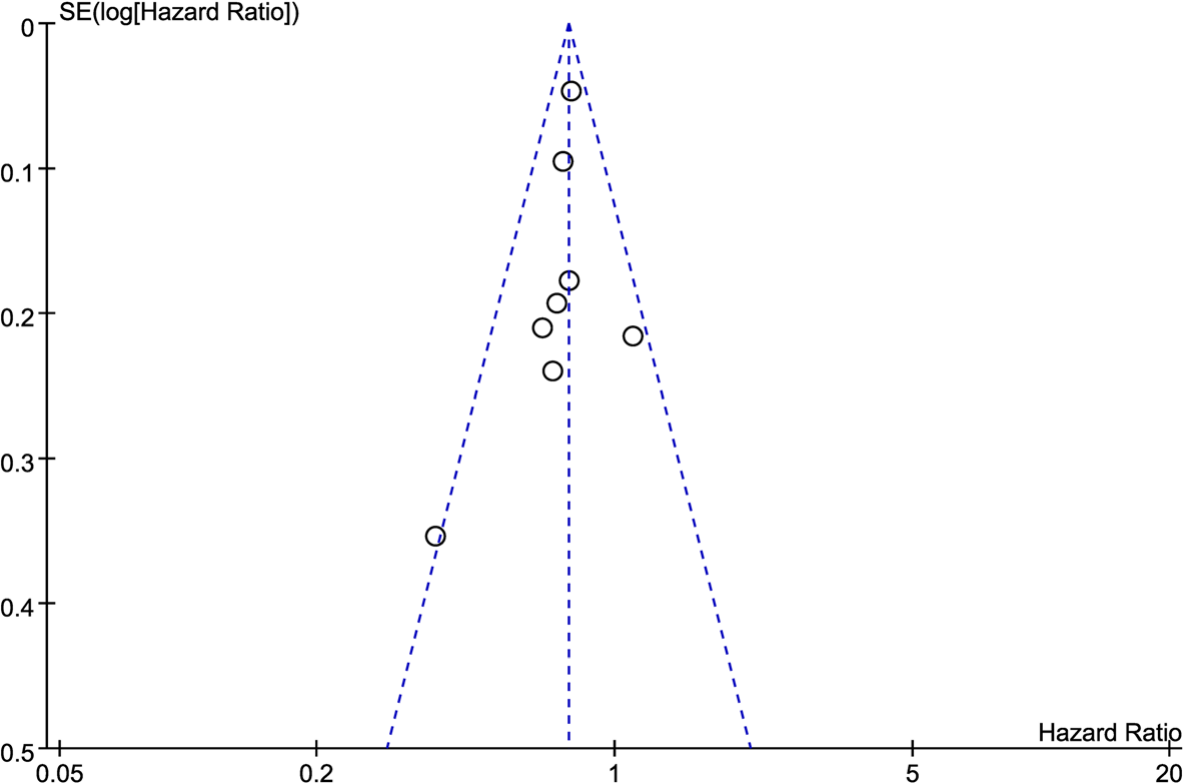
Funnel Plot for publication bias of selected meta-analyses

Publication bias statistical analysis was performed using the Funnel Plot. As shown in Fig. 6, no publication bias was detected in meta-analysis of HD-RT vs SD-RT (*P* = 0.38, I2 = 6%).

A summary of findings table is presented in Additional file 1. We used the GRADE approach for this meta-analysis to appraise the confidence in estimates. In line with GRADE guidelines, the non-randomized studies started as low quality due to residual confounding. Furthermore, publication bias, an overall large effect and a dose response gradient were not identified. Thus, the studies were deemed to be of low quality.

## Discussion

Definitive CCRT is considered as the optimal choice for patients with non-operable EC, especially ESCC. However, the standard dose of RT still remains controversial. On the basis of results from RTOG 94–05, 50.4 Gy has been accepted as standard dose in western countries and recommended by NCCN guideline for more than a decade [6]. Based on the theory of radiation biology, 50.4Gy is just adequate to control microscopic cancer cell, but inadequate to control a gross tumor lesion [19]. A radiation dose more than 60Gy or even nearly 100Gy is required to control and cure a gross solid tumor [19]. According to statistics, only a few patients with EC received a radiation dose of 50.4Gy would achieve complete response and obtain a long term survival [20]. Although RTOG 94–05failed to show evidence of increased dose could improve LC and OS, several retrospective studies have reported that increased radiation dose could improve local control and OS by enhancing clinical complete response rate. A pooled analysis from Song et al. showed that a higher radiation dose could improve clinical outcomes without significantly increasing radiation-related toxicities, which was contradictory to RTOG 94–05 [8]. However, this review was a compilation of single treatment arm, with only 3 included studies containing both a HD-RT (≥ 60 Gy) arm and SD-RT arm. Another meta-analysis has indicated that patients who received ≥60 Gy radiation had a significantly better prognosis as compared with < 60 Gy [21]. The studies including patients received < 50 Gy radiation and the studies including patients diagnosed as esophageal adenocarcinoma were included in the meta-analysis. In this present study, we selected studies which contained both HD-RT and LD-RT arms and studies in which the patients received radiotherapy delivered by modern radiotherapy technique such as 3D-RT and IMRT to reduce bias and heterogeneity. We excluded studies those mainly included patients with esophageal adenocarcinoma and patients received < 45 Gy radiation. Our study indicated that HD-RT improve OS, PFS and LRFS as compared to SD-RT. With this meta-analysis, we supported that patients with non-operable ESCC should be treated with a higher dose radiation of ≥60 Gy in clinical practice, as well as hoped that further randomized control trial comparing HD-RT with SD-RT delivered by modern radiation techniques in specific ESCC population would be carried out in our country.

In RTOG 94–05 and previous retrospective studies, RT was delivered by 2DRT or Co60 radiation which was now-outdated technique and may bring about risk of radiation toxicity in lung and heart [6, 22]. With the clinical application of more precise radiation techniques such as 3DRT or IMRT, interpretation about the results of RTOG 94–05 should be different. 3DRT and IMRT might provide radiation dose escalation to the gross tumor volume while reducing the dose to the organs at risk [23]. In the RTOG0436 trial, a V20 limit of < 25% would reduce the incidence of grade 3–4 dyspnea with only 1.6% (5/319) in the CCRT arm with 3D-CRT [24]. Hsieh et al. reported that no patient suffered from symptomatic pneumonitis using IMRT to a total dose> 50.4 Gy in a series of 29 patients with locally advanced EC [25]. These modern techniques allow the radiation oncologist to deliver higher doses of radiation with less toxicity to the surrounding normal tissue, prompting oncologist to investigate whether dose escalation based on modern radiation techniques improve patients outcomes safely, especially for ESCC patients. A phase II study from Chen and his colleague have demonstrated that radiation dose escalation using simultaneous modulated accelerated radiotherapy (SMART) combined with concurrent chemotherapy was feasible in ESCC patients with tolerable acute toxicities [11]. However, there is lack of a randomized control trial to investigate whether higher radiation dose using modern radiation technique improve outcome of ESCC patients with tolerable toxicities. In this study, we provided an up-to-date, reliable, and comprehensive summary of the effect of HD-RT compared to SD-RT in patients received 3DRT and IMRT, which would give us some implications for clinical practice and future research.

According to radiation biological theory, a radiation dose of 60 Gy or higher is needed to abrogate a gross solid tumor [19]. Therefore, 60Gy was frequently used as a threshold between high dose and standard dose in many studies when investigating the effect of radiation dose escalation in non-operable EC patients [13, 26]. However, in some studies, especially studies from western countries, one value between 50Gy and 60Gy was used as the threshold value for high dose and standard dose, which would bring about dose heterogeneity when a meta-analysis was performed [27, 28]. In this present study, we defined a radiation dose of > = 60 Gy as HD-RT and < 60 Gy as SD-RT. Furthermore, only those studies using 60Gy as a threshold value for high dose and standard dose were included in our meta-analysis, and we have found that high radiation dose of > = 60 Gy was associated with better OS, PFS and LRFS, which is consistent with Chen’s study and Song’s study [8, 21]. However, how high radiation dose is appropriate for ESCC patients? According to Chen’s study, a dose of ≥60 Gy could improve patients’ OS compared with a dose of < 60 Gy, but an extremely high radiation dose of 70 Gy did not result in extra benefit or clinical outcome. In a Phase II dose-escalation study, Chen et al. indicated that a simultaneous integrated boost radiotherapy (RT consisted of 66 Gy at 2.2 Gy per fraction to the gross tumor) combined with concurrent chemotherapy is feasible in EC patients with tolerable acute toxicities [11]. In their study, only 5 of them (8.4%) had local recurrence, while there were already 2 cases (3.3%) of treatment-related death due to esophageal hemorrhage. Another recently published Phase I dose-escalation study, Yu et al. suggest that it is feasible to deliver up to 70 Gy (2.8Gy/F) to the GTV [10]. However, no information of late toxicities was provided in this study. Thus, a radiation dose between 60Gy and 70Gy may be optimal and further dose-escalation may lead to increased risk of treatment-related death, rather than clinical benefit.

Inevitably, there are some limitations in this study. Firstly, most of the studies included were retrospective studies except one RCT and one population based propensity-score matched analysis. Secondly, several studies had a relative small amount of patients in each group. Thirdly, there were significant heterogeneities in PFS and LRFS meta-analysis. Fourthly, some specific information, such as chemotherapy regimens and radiotherapy field design, were unable to be obtained in the included studies, hence we could not conduct subgroup analyses based on these factors. Last but not the least, due to non-randomized approach to treatment selection, there must be some potential unmeasured selection biases regarding the patient physical and organ status, same clinical stage of disease but more extensive disease, preference of radiation oncologist, or other patient-related factors. These limitations might have influenced our findings and will reduce the effectiveness of the existing clinical evidence. Indeed, more RCTs are needed to further support our conclusions.

## Conclusion

In conclusion, we performed an up-to-date and comprehensive summary of the effect of HD-RT and SD-RT in ESCC patients who received modern radiotherapy and our results suggest that HD-RT (a higher radiation dose of > = 60 Gy) could bring about better locoregional control and OS than SD-RT therapy. In the future, Phase III trials comparing the effect and toxicity of SD-RT and HD-RT using modern RT technique are warranted in the right subgroup population of ESCC patients.

## Supplementary information

**Additional file 1.** Summary of Finding Table.

## Data Availability

The datasets used and/or analyzed during the current study are available from the corresponding author upon reasonable request.

## Abbreviations

3DRT: Three dimensional conformal radiotherapy
CCRT: Definitive concurrent chemoradiotherapy
CI: Confidence interval
CTV: Clinical target volume
CTVnd: Nodal clinical target volume
CTVt: Tumor clinical target volume
EAC: Esophageal adenocarcinoma
EC: Esophageal cancer
ESCC: Esophageal squamous cell cancer
GTV: Gross tumor volume
GTVnd: Nodal gross tumor volume
HD-RT: High-dose radiotherapy
IMRT: Intensity-modulated radiotherapy
LC: Local control
LRFS: Local recurrence-free survival
OS: Overall survival
PFS: Progression-free survival
PTV: Planning target volume
SD-RT: Standard-dose radiotherapy
