# Supplementary material for: Effect of modern high-dose versus standard-dose radiation in definitive concurrent chemo-radiotherapy on outcome of esophageal squamous cell cancer: a meta-analysis
Source: Radiat Oncol. 2019 Oct 17;14:178. doi: 10.1186/s13014-019-1386-x (PMC6798457; doi:10.1186/s13014-019-1386-x)
Supplement: Supplementary file 1 — Additional file 1. Summary of Finding Table. [file 13014_2019_1386_MOESM1_ESM.doc]

| **Supplementary Table Summary of Finding Table**  **Topic: Standard dose CCRT compared to High dose CCRT for Esophageal cancer** | | | | | | |
| --- | --- | --- | --- | --- | --- | --- |
| **Patient or population:** patients with Esophageal cancer **Settings:** Chemoradiotherapy **Intervention:** High dose CCRT **Comparison:** Standard dose CCRT | | | | | | |
| **Outcomes** | **Illustrative comparative risks (95% CI)** | | **Relative effect (95% CI)** | **No of Participants (studies)** | **Quality of the evidence (GRADE)** |  |
|  | **Standard dose CCRT** | **High dose CCRT** |  |  |  |  |
| **Overall survival**  Hazard Ratio Follow-up: 1-164.7 months | Not estimable | Not estimable | HR ranged from 0.72 to 0.84 | 3664 (8 studies) | ⊕⊕⊝⊝ **low** |  |
| **2-year survival rate** Relative Risk Follow-up: 1-164.7 months | **297 per 1000** | **372 per 1000** (339 to 407) | **RR 1.25**  (1.14 to 1.37) | 3664 (8 studies) | ⊕⊕⊝⊝ **low** |  |
| **Progression-Free survival**  Hazard Ratio Follow-up: 2.2-164.7 months | Not estimable | Not estimable | HR ranged from 0.57 to 0.87 | 588 (4 studies) | ⊕⊕⊝⊝ **low** |  |
| **Local recurrence-free survival** Hazard Ratio Follow-up: 2.2-164.7 months | Not estimable | Not estimable | HR ranged from 0.36 to 0.74 | 362 (2 studies) | ⊕⊕⊝⊝ **low** |  |
| **CI:** Confidence interval; **HR:** Hazard ratio;**RR:** Relative Risk. | | | | | | |
|  | | | | | | |
